# Evaluation of adverse events and comorbidity exacerbation following the COVID-19 booster dose: A national survey among randomly-selected booster recipients

**DOI:** 10.1371/journal.pone.0326231

**Published:** 2025-07-11

**Authors:** Dvora Frankenthal, Miri Zatlawi, Ziv Karni-Efrati, Lital Keinan-Boker, Aharona Glatman-Freedman, Michal Bromberg

**Affiliations:** 1 Ministry of Health, The Israel Center for Disease Control, Ramat Gan, Israel; 2 Center for Research and Study of Aging, University of Haifa, Haifa, Israel; 3 School of Public Health, University of Haifa, Haifa, Israel; 4 The school of Public Health, Faculty of Medical and Health Sciences, Tel Aviv University, Tel Aviv, Israel; Stanford University School of Medicine, UNITED STATES OF AMERICA

## Abstract

**Background:**

Periodic vaccination against COVID-19 persists with a recommendation to vaccinate especially older people and the chronically ill. However, vaccination compliance is low, likely due to concerns regarding adverse events (AEs).

**Objective:**

To systematically and proactively evaluate the occurrence, onset, duration, and severity of self-reported AEs and comorbidities exacerbations that appeared up to 21–30 days following the third (booster) Pfizer BNT162b2 vaccine dose, and to examine the associations between the occurrence of any AEs and sociodemographic and pre-existing comorbidities.

**Methods:**

A cross-sectional telephone survey among a nationally representative sample of Israeli vaccinated adults aged ≥18 was conducted from September through October 2021. Sociodemographic data was extracted from the Ministry of Health vaccination database, and data on AEs and comorbidities were collected using a structured questionnaire.

**Results:**

Overall, 2,049 participants completed the survey (71.4% response rate). A total of 1360 (66.4%) reported at least one AE following the booster vaccine. The most frequently reported AEs were local (55.7%) and mild systemic (48.6%) reactions (i.e., fatigue, headache, fever), followed by neurological (4.5%) and allergic (3.9%) reactions. Exacerbation of comorbidities following the booster dose was most frequently reported by individuals with autoimmune or mental conditions. Most local (80.1%) and systemic (69.5%) reactions lasted up to three days. Only 8.3% sought medical care. Menstrual changes were reported by 9.6% of women aged <54 years. The occurrence of any AEs was associated with younger age, female gender, higher socioeconomic status, and living in suburban communities. AEs were not associated with pre-existing comorbidities.

**Conclusion:**

Most AEs were mild to moderate and transient. They were associated with younger age, but not with pre-existing chronic diseases. Since the primary target population for vaccination consists of older individuals and those with comorbidities, we believe the current findings may assist in reducing COVID-19 vaccine hesitancy among these populations.

## Introduction

Safety is paramount for vaccine acceptance and use. In December 2020, Israel initiated a mass vaccination campaign against SARS-CoV-2 with the Pfizer-Biontech® BNT162b2 vaccine [1]. By May 2021, approximately 80% of Israel’s adult population had received two doses of the vaccine [2]. With continuing circulation of SARS-CoV-2 changing variants, repeated vaccination is recommended, primarily for people ≥65 years old and those with pre-existing medical conditions [3]. In October 2024, the US Centers for Disease Control and Prevention (CDC), recommended that individuals ≥65 years old and those who are moderately or severely immunocompromised receive two doses of the 2024−2025 COVID-19 vaccine, six months apart [4].

However, compliance with the booster vaccines, in many countries, has been lower than that of the primary COVID-19 vaccine doses [5,6]. Specifically, while the mean reported vaccination rates for the first two doses was 75.2% (standard deviation, SD, 13.4%), the mean reported rate for the booster dose was 44.9% (SD 17.2%) in a sample of countries from around the world [5]. The main reasons reported for vaccine and booster hesitancy included insufficient safety data, fear of the vaccine’s adverse events (AEs), altered perception of disease risk, and distrust of government [5].

In various countries across the globe, including Israel, vaccine AEs are monitored mostly by voluntary passive reporting to a vaccine AEs reporting system done by either healthcare workers or the vaccinees themselves [7–14]. However, AEs recorded in these systems have been prone to underreporting [15]. During the COVID-19 pandemic, other platforms for AE reporting were used in the United States [16]. Some platforms were voluntary active mobile telephone-based, while others relied on medical claims or clinical studies [16]. As repeated COVID-19 booster vaccines are likely to be recommended, it is important to provide comprehensive information about potential AEs, to reduce hesitancy. Recent post-marketing studies on AEs following booster doses of mRNA COVID-19 vaccines used passively reported data (such as Vaccine Adverse Event Reporting System, VAERS) [14,17–19], voluntary active mobile telephone-based surveillance system [19], self-controlled case series methods [20,21], or evaluated only specific AEs [17,18,22].

To our knowledge, to date, there is limited data on self-reported AEs after the mRNA COVID-19 booster vaccine of vaccinated adults who were approached systematically and proactively. Therefore, this survey aimed to systematically and actively examine the occurrence, onset, duration, and severity of self-reported AEs, and comorbidities exacerbations up to 21–30 days after the third (booster) dose of the Pfizer-Biontech® BNT162b2 vaccine among a nationally representative sample of Israeli vaccinated adults. A secondary aim was to examine the associations between the occurrence of any AEs and sociodemographic and pre-existing comorbidities.

## Materials and methods

### Survey population

This national cross-sectional telephone survey was conducted between September 19 and October 25, 2021, by the Israel Center for Disease Control (ICDC). A random sample of telephone numbers of vaccinated adults was extracted from the Ministry of Health repository of COVID-19 vaccinations using SAS statistical software version 9.4 (Cary, NC, USA). Each COVID-19 vaccine administered in Israel was individually recorded in the repository with data regarding the vaccinee [1]. The random sample was stratified by sex and age groups (18−39, 40−59, and ≥60 years) of the vaccinee. The sample included community-dwelling adults aged ≥18 who received the BNT162b2 booster dose 21−30 days before the survey. Participants were excluded from the sample if they were documented as SARS-CoV-2-positive at any time before the survey, or did not speak Hebrew. SARS-CoV-2 status was determined based on documentation in the national SARS-CoV-2 tests database [1,23]. The interviews were conducted in Hebrew using a Computer Assisted Telephone Interview (CATI) system.

### Questionnaire

A structured and comprehensive questionnaire was developed by the ICDC.

The questionnaire aimed to assess the occurrence**,** onset time, duration, and severity of AEs following the booster vaccine dose.

AEs were evaluated by asking respondents whether they had any AE after the third vaccine dose. If they answered positively, they were then asked about any local reactions using a structured list. Participants who reported AEs not included in the list were invited to describe them in their own words.

The onset and duration of local reactions were categorized into specific time intervals.

The severity of the local reactions was measured by inquiring whether respondents experienced difficulties in regular daily activities, sought medical care, and/or were hospitalized.

Comparison to local reactions after previous doses of the vaccine was assessed by asking the respondents whether similar reactions appeared either after the first or the second COVID-19 vaccine dose. If the answer was positive, respondents were asked to qualify whether those were milder, worse, or of similar severity.

A similar set of questions was repeated for systemic (i.e., fatigue, headache, myalgia/ arthralgia, chills, fever, gastrointestinal symptoms, dizziness, chest pain, lymphadenopathy, cough, anxiety, other), allergic (i.e., itching, dyspnea, rash, swelling of face or throat, other), neurological (i.e., vision disorders, hearing disorders, memory loss, seizures, paresthesia, Bell’s palsy, syncope, other) and other reactions (i.e., herpes zoster, herpes simplex, menstrual changes among women aged <54 years, other). If the answer was positive for menstrual changes, participants were asked to describe the changes and to report whether they had menstrual cycle irregularities before the first COVID-19 vaccine. Women who reported changes following the booster were contacted again four months post-vaccination and asked whether their period cycle had returned to the regular pattern and when (one-/2-/3-/4-months post-vaccination/not to date).

Prevalence of comorbidities (i.e., hypertension, lung disease, diabetes, heart disease, depression and/or anxiety, autoimmune disease) and whether they had exacerbated following the booster dose were also assessed.

### Sociodemographic variables

The following sociodemographic variables were obtained from the COVID-19 vaccine database: age, sex, socioeconomic status (based on the statistical geographic area of the respondents’ residence), and residence. These variables were grouped by age (18–39, 40–59, ≥ 60 years of age), sex (female, male), socioeconomic status (low, medium, high), and type of residence (city, local council, suburban community).

### Sample size

The prevalence of systemic AEs after the booster COVID-19 vaccine was estimated as 25% which aligns with the rate reported in the literature for systemic AEs after the BNT162b2 second booster vaccine [24]. The margin of error and confidence level were defined as 2.0 and 95% respectively. To meet these criteria, the minimum recommended sample size was determined to be 1,800 participants. This sample size was calculated using Epi Info, an open-source calculator (https://www.cdc.gov/epiinfo/index.html).

### Statistical analysis

Bivariate analyses was performed using a Pearson’s Chi-square test to compare sociodemographic and health-related characteristics between participants who reported any AEs and those who did not. A multivariate logistic regression model was applied to determine the associations between the occurrence of any AEs and participants’ characteristics. Sociodemographic and health-related variables such as age, sex, socioeconomic status, residence, and the presence of comorbidity were entered into the multivariate logistic regression model only if they demonstrated a significance level of p < 0.1 in bivariate analyses. A p-value of <0.05 was considered statistically significant. Data were analyzed using SAS statistical software version 9.4 (Cary, NC, USA).

### Ethics statement

Ethical approval for the study was waived by the National Ethical Committee for Human Medical Research of the Israel Ministry of Health. The committee determined that the survey was part of the Ministry of Health’s official activities. Verbal informed consent was obtained from each participant after a brief explanation of the health survey, including its objectives and significance. The consent to participate was documented in the questionnaire. All data collected were coded anonymously to ensure confidentiality.

## Results

### Survey population

A random sample of 4,945 telephone numbers of Israeli citizens vaccinated with a third dose of the BNT162b2 vaccine 21–30 days ago was extracted. After applying the survey’s exclusion criteria, a total of 2,894 people remained eligible to participate. Of them, 2,068 participants were interviewed (response rate of 71.4%). After excluding 19 inconsistent interviews, the sample for statistical analysis included 2,049 participants (S1 Table). The average age was 47.7 ± 16.7 (years±SD) and about half (49.1%) of the participants were females. To assess potential non-response bias, we compared the sociodemographic characteristics of survey respondents with those of non-respondents. Our analysis revealed no differences in age, sex, and type of residence between non-respondents and respondents (S2 Table). However, a higher percentage of individuals with lower socioeconomic status did not respond to the survey compared to those who did respond (24.4% vs. 16.4%).

Table 1 presents the sociodemographic and health-related characteristics of the study participants by AE occurrence. A total of 1,360 (66.4%) participants reported at least one AE following the booster vaccine. AEs were more prevalent among younger ages, females, individuals with higher socioeconomic status, and participants without pre-existing comorbidities.

**Table 1:** Sociodemographic characteristics and pre-existing comorbidities among survey participants (N = 2,049) by adverse events occurrence.

Variable	Total N = 2049 N (%)	Adverse events N = 1360 N (%)	No adverse events N = 689 N (%)	P-value
**Age groups (years)**				
18-39	653 (31.9)	466 (34.3)	187 (27.1)	<0.001
40-59	751 (36.6)	525 (38.6)	226 (32.8)	
60+	645 (31.5)	369 (27.1)	276 (40.1)	
**Sex**				
Male	1,044 (50.9)	602 (44.3)	442 (64.1)	<0.001
Female	1,005 (49.1)	758 (55.7)	247 (35.9)	
**Residence**				
City	1,539 (75.1)	1,004 (73.8)	535 (77.7)	<0.01
Local council	276 (13.5)	180 (13.2)	96 (13.9)	
Suburban community	234 (11.4)	176 (12.9)	58 (8.4)	
**Socioeconomic category status**				
Low	326 (16.4)	184 (13.9)	142 (21.4)	<0.001
Medium	1,061 (53.4)	715 (54.1)	346 (52.0)	
High	599 (30.2)	422 (32.0)	177 (26.6)	
Missing (N = 63)				
**Comorbidities** ^1^				
Yes No Missing (N = 51)	659 (33.0) 1339 (67.0)	406 (30.8) 912 (69.2)	253 (37.2) 427 (62.8)	<0.05
**Hypertension**				
Yes No Missing (N = 32)	285 (14.1) 1732 (85.9)	170 (12.8) 1162 (87.2)	115 (16.8) 570 (83.2)	<0.05
**Lung disease**				
Yes No Missing (N = 15)	201 (9.9) 1833 (90.1)	130 (9.6) 1218 (90.4)	71 (10.4) 615 (89.6)	0.614
**Diabetes**				
Yes No Missing (N = 31)	151 (7.5) 1867 (92.5)	77 (5.8) 1260 (94.2)	74 (10.9) 607 (89.1)	<0.001
**Heart disease**				
Yes No Missing (N = 22)	110 (5.4) 1917 (94.6)	60 (4.5) 1282 (95.5)	50 (7.3) 635 (92.7)	<0.01
**Depression and/or anxiety**				
Yes No Missing (N = 26)	91 (4.5) 1932 (95.5)	63 (4.7) 1276 (95.3)	28 (4.1) 656 (95.9)	0.530
**Autoimmune Disease**				
Yes No Missing (N = 25)	62 (3.1) 1962 (96.9)	39 (2.9) 1303 (97.1)	23 (3.4) 659 (96.6)	0.565

^1^ Comorbidities included hypertension and/or lung disease and/or diabetes and/or heart disease and/or depression and/or anxiety and/or autoimmune disease.

### Adverse events

Table 2 shows that the most frequently reported AEs were local (55.7%), followed by systemic (48.6%), neurological (4.5%) and allergic (3.9%) reactions. A total of 59 (9.6%) of the 615 women aged <54 years reported menstrual changes following the booster dose. One patient (0.05), a 43-year-old female, reported myocarditis.

**Table 2:** Adverse events (AEs) after the BNT162b2 booster dose reported by survey participants (N=2,049).

AEs Category	AE	Participants [N (%)]
**Any**		**1,360 (66.4)**
Local reactions	Any	1,140 (55.7)
	Pain	1,108 (54.2)
	Limitation in arm mobility	473 (23.3)
	Swelling	257 (12.6)
	Enlarged lymph nodes	185 (9.1)
	Redness	131 (6.5)
Systemic reactions	Any	995 (48.6)
	Fatigue	856 (42.5)
	Headache	529 (26.3)
	Myalgia/Arthralgia	520 (25.7)
	Chills	344 (17.1)
	Fever (>38.0^0^C)	306 (15.2)
	Gastrointestinal	191 (9.6)
	Dizziness	186 (9.3)
	Chest pain	110 (5.5)
	Lymphadenopathy	84 (4.2)
	Cough	78 (3.9)
	Anxiety	41 (2.0)
	Other^1^	40 (2.0)
Neurological reactions	Any	91 (4.5)
	Paresthesia	68 (3.4)
	Bell’s palsy	11 (0.5)
	Vision disorder/blurred vision	11 (0.5)
	Memory loss	8 (0.4)
	Hearing disorder	7 (0.4)
	Seizures	4 (0.2)
	Syncope	3 (0.2)
Allergic reactions	Any	80 (3.9)
	Rash	40 (2.0)
	Itching	34 (1.7)
	Dyspnea	30 (1.5)
	Swelling of face or throat	13 (0.6)
Other AEs	Any	83 (4.1)
	Herpes simplex	4 (0.2)
	Herpes zoster	3 (0.2)
	Menstrual changes among women <54 years (N = 615)^2^	59 (9.6)
	Myocarditis	1 (0.05)
	Other^2^	21 (1.0)

^1^ “Other” included sore throat, nasal congestion, swelling/heaviness of legs, mouth sores, low-grade fever, hair loss, hot flashes, restlessness;

^2^ “Other” included eye disorders, change in smell/taste.

Table 3 demonstrates that most local and systemic reactions occurred within the first 24 hours after vaccination and lasted up to three days. On the other hand, neurological, allergic, and other AEs, that were less frequently reported, occurred in substantial proportions also later on, including 1−4 weeks post-vaccination, with many symptoms still prevalent at the time the interview took place (21−30 days following the booster vaccine). Moreover, 9.4%−21.1% of participants who reported AEs described worse AEs after the third vaccine dose, as compared to previous doses.

**Table 3:** Onset, duration, and severity of adverse events after the BNT162b2 booster dose by type of adverse reaction.

	Local reactions N (%)	Systemic reactions N (%)	Neurological reactions N (%)	Allergic reactions N (%)	Other adverse events N (%)
**Onset of adverse event**					
Within 24 hours Within 1–7 days Within 1–4 weeks Missing (N)	858 (76.1) 259 (23.0) 10 (0.9) 13	583 (59.8) 327 (33.5) 65 (6.7) 20	35 (42.2) 24 (28.9) 24 (28.9) 8	26 (35.1) 26 (35.1) 22 (29.7) 6	7 (14.3) 19 (38.8) 23 (46.9) 34
**Duration of adverse event**					
Up to 24 hours Between 1–3 days Between 4–7 days >7 days Has not resolved to date Missing (N)	269 (23.8) 636 (56.3) 158 (14.0) 42 (3.7) 24 (2.1) 11	261 (26.8) 415 (42.7) 115 (11.8) 58 (6.0) 124 (12.7) 22	19 (21.3) 18 (20.2) 7 (7.9) 3 (3.4) 42 (47.2) 2	7 (9.2) 19 (25.0) 14 (18.4) 11 (14.5) 25 (32.9) 4	1 (2.2) 6 (13.3) 8 (17.8) 11 (24.4) 19 (42.2) 38
**Severity in comparison to similar adverse events after previous vaccine doses**					
Milder Worse Similar Not applicable^1^ Missing (N)	157 (14.0) 237 (21.1) 478 (42.5) 252 (22.4) 16	149 (15.3) 165 (16.9) 245 (25.2) 415 (42.6) 21	5 (5.6) 11 (12.4) 18 (20.2) 55 (61.8) 2	5 (6.7) 15 (20.0) 8 (10.7) 47 (62.7) 4	1 (1.9) 5 (9.4) 15 (28.3) 32 (60.4) 30

^1^ There were no similar adverse events after previous vaccine doses.

Multivariate logistic regression analysis (Table 4) showed that reporting AEs was associated with younger age, female sex, higher socioeconomic status, and living in suburban communities. AEs were not associated with the presence of pre-existing comorbidities.

**Table 4:** Adjusted odds ratios (ORs) for the associations between participants’ characteristics and occurrence of an adverse reaction after the booster dose.

Multivariate analysis			
**Variable**	**OR**	**95% CI**	**P value**
**Age**			
60+ 45-59 18-39	1 (Ref) 1.83 1.97	1.43-2.34 1.51-2.57	<0.001 <0.001
**Sex**			
Male Female	1 (Ref) 2.25	1.85-2.74	<0.001
**Socioeconomic status**			
Low Medium High	1 (Ref) 1.48 1.58	1.14-1.93 1.17-2.12	<0.01 <0.01
**Residence**			
City Local council Suburban community	1 (Ref) 0.96 1.67	0.72-1.27 1.19-2.34	0.757 <0.01
**Comorbidities** ^1^			
No Yes	1 (Ref) 0.99	0.79-1.24	0.953

CI = confidence interval; OR= odds ratio;

^1^ Comorbidities included hypertension and/or lung disease and/or diabetes and/or heart disease and/or depression and/or anxiety and/or autoimmune disease.

### Exacerbation of comorbidities

Table 5 demonstrates comorbidities exacerbations following the booster dose reported by participants with comorbidities. Exacerbations were most commonly reported by participants with depression and/or anxiety (26.4%) followed by participants with autoimmune diseases (24.2%). Exacerbations of other comorbidities were reported in less than 10% of participants for each specific comorbidity.

**Table 5:** Comorbidities among survey participants and their exacerbations following the booster dose.

Comorbidities	Participants (N = 2,049) [N (%)]	Exacerbation following the booster dose [N (%)]^1^
Hypertension	285 (14.1)	18 (6.3)
Lung disease	201 (9.9)	14 (7.0)
Diabetes	151 (7.5)	14 (9.3)
Heart disease	110 (5.4)	6 (5.5)
Depression and/or anxiety	91 (4.5)	24 (26.4)
Autoimmune Disease	62 (3.1)	15 (24.2)

^1^ Calculated from those who reported the specific comorbidity.

### Health impacts

Among participants with any AE, 589 (44.1%) reported difficulties in regular daily activities due to the AE. However, only 107 (8.3%) sought medical care and 6 (0.5%) were hospitalized (Table 6). Participants who reported having both local and non-local AEs constituted the largest group of those reporting health impacts (Table 6). Among the hospitalized patients, two were hospitalized with chest pain, and two had syncope and dyspnea. The patient who reported myocarditis reported being referred to the emergency department but was not subsequently hospitalized.

**Table 6:** Health impact among participants who reported adverse events by type of AEs.

AEs’ health impact	Responded N = 1,360 N (%)^1^	Any AEs N (%)^2^	Local AEs N = 327 N (%)	Non-local AEs N = 220 N (%)	Both types N = 813 N (%)	p-value
Difficulties in regular daily activities	1,337 (98.3)	589 (44.1)	38 (2.8)	93 (7.0)	458 (34.2)	<0.001
Sought medical care	1,286 (94.6)	107 (8.3)	2 (0.2)	21 (1.6)	84 (6.5)	<0.001
Hospitalized	1,342 (98.7)	6 (0.5)	1 (0.1)	1 (0.1)	4 (0.3)	0.917

^1^ Percentage calculated from1360 respondents.

^2^ Percentage calculated from respondents for each type of health impact.

### Menstrual cycle changes

Of 59 (9.6%) women aged <54 years who reported menstrual changes following the booster dose, only 45 described the menstrual cycle changes. Table 7 shows that the main reported menstrual cycle changes were delayed onset of menstruation (N = 21, 46.7%), heavy menstrual bleeding (N = 15, 33.3%), and earlier menstruation (N = 13, 28.9%). Most women (N = 39, 88.6%) reported no menstrual cycle irregularities before the first COVID-19 vaccine dose. About a third of women (N = 16) reported that they had menstrual cycle irregularities also after the first or second COVID-19 vaccine. A follow-up telephone contact four months after the booster dose was successfully established with 41 women. In approximately a quarter of the women (N = 11, 26.8%) the menstrual cycle returned to normal after one cycle, in 5 (12.2%) and 4 (9.8%) of them, the menstrual cycle returned to normal after two and three months, respectively. However, in approximately half (N = 21, 51.2%) of the women, the menstrual changes remained four months post-vaccination.

**Table 7:** Changes in menstrual cycle after the BNT162b2 booster dose among women<54 years (n = 45).

Menstrual changes^1^	n (%)
Delay in menstruation	21 (46.7)
Heavy menstrual bleeding	15 (33.3)
Earlier menstruation	13 (28.9)
Duration of bleeding longer than usual	12 (26.7)
Multiple bleedings during the month following the vaccine	11 (24.4)
Menstrual cramps	9 (20.0)
Lighter menstrual bleeding	4 (8.9)
Duration of bleeding shorter than usual	2 (4.4)
Unexpected vaginal bleeding after vaccination	1 (2.2)
Blood clots	1 (2.2)
Recurrence of menstruation after amenorrhea	1 (2.2)

^1^ More than one menstrual change could be reported by each respondent.

## Discussion

This survey was conducted by proactively reaching out to booster recipients via telephone. This approach differed from most post-marketing reports of COVID-19 vaccine AEs, which typically relied on passive reporting systems such as VAERS [19,25–27] or the Yellow Card [28]. Furthermore, to the best of our knowledge, most published studies of COVID-19 vaccine AEs to date relied on data either from the passive reporting systems [25–27] or from electronic health records [20,21]. The advantage of our data collection method is that it increases the likelihood of receiving responses from individuals who would not voluntarily report AEs, as well as from those whose AEs did not require medical care.

Furthermore, our survey’s stratification by sex and age groups enabled an equal distribution of female and male respondents across various age groups. This is in contrast to studies that relied on passive reporting of the booster vaccine’s AEs, where the number of female respondents was nearly double that of male respondents [25,27]. Additionally, one study [24] indicated 6-fold more AE reporters among <65 years old as compared to those 65 years and older, while another study [26] found that the number of AE reporters aged 65 and older was about 30% higher than in other adult age groups.

In this survey, we found that most AEs following booster vaccination were transient local or general systemic reactions lasting up to three days. Among participants experiencing any AE, only a small percentage (8.3%) sought medical care indicating that most AEs were not severe.

AEs were less common among participants ≥60 years old and were not associated with pre-existing comorbidities. While the finding that older participants had fewer AEs was consistent with other studies on AEs following the booster dose [25,27], we could not find published data regarding the specific association between COVID-19 booster dose AEs and pre-existing comorbidities. These findings are particularly significant as SARS-CoV-2 continues to circulate and cause disease globally, and the main target populations for periodic COVID-19 boosters, are older individuals and those with pre-existing comorbidities [29]. Counseling on AEs by health care providers is strongly recommended as our findings could assist in reducing the barriers to receiving a booster vaccine by these specific populations. Indications for a higher incidence of AEs among women and younger adults, as shown in the current survey, are consistent with other studies [25,27]. Scholars argued that the occurrence of AEs following BNT162b2 vaccination is associated with enhanced antibody response [30]. In this regard, higher titers of antibodies found among vaccinated females can explain their higher frequency of post-vaccination AEs, compared with males [31,32]. Similarly, the lower antibody response found among older adults vaccinated with the BNT162b2 vaccine may explain the lower frequency of AEs reported by them.

In contrast to local and systemic reactions, allergic and neurological reactions reported in our survey, were infrequent, had a more delayed onset and a longer duration.

In a significant number of participants, these reactions lasted even longer than 21–30 days following the booster dose. It is recommended to raise awareness among health care providers about long-term adverse effects following booster vaccinations.

Regarding patients with comorbidities, our findings indicated that nearly a quarter of the participants with either depression/anxiety or an autoimmune disease reported an exacerbation of their comorbidity following the booster vaccine dose. Deterioration of a psychiatric disease during the COVID-19 pandemic was reported among more than half of the patients with this comorbidity [33,34]. Furthermore, COVID-19 vaccination was found to be associated with a lower incidence of mental illness following COVID-19 disease, as compared with unvaccinated individuals [35]. Thus, the rate of anxiety/depression following the COVID-19 booster reported in our study is likely lower than expected following COVID-19 disease. Flare-ups of autoimmune diseases following COVID-19 vaccination were previously reported. For example, they were reported by 11.3% to 26.7% of patients with autoimmune rheumatic disease [36], and by 5% to 6% of patients with systemic lupus erythematosus [37]. Therefore, it is important to counsel and monitor people with autoimmune and mental diseases after a COVID-19 booster vaccination.

Special emphasis was given in the current survey to changes in the menstrual cycle among women. Approximately 10% of women under the age of 54 reported experiencing menstrual changes after receiving the booster vaccine, with most of them not experiencing such changes following the first or second vaccine doses.

There are currently relatively few studies that address menstrual changes following the booster vaccine. A study from Japan suggested that the COVID-19 booster can be associated with more prolonged menstrual cycles and longer persistence of menstrual changes compared to the first and second vaccine doses [38]. In this regard, it is important to note that menstrual changes were reported also following COVID-19 disease [39]. An online nationwide survey among 10,319 premenopausal non-pregnant Israeli women found that the type of menstrual disturbances was similar among COVID-19-vaccinated and infected women, primarily characterized by excessive bleeding [40]. These results were recently supported by a multinational study [39]. Our findings and those of others, highlight the necessity to counsel women about what to expect following the COVID-19 booster.

Only one case of myocarditis, which appeared to be mild, was reported following the booster dose. Cases of myocarditis were previously reported following the COVID-19 mRNA vaccine booster dose [41,42], However, based on studies that relied on VAERS, myocarditis was reported in lower numbers following booster doses as compared with the second dose [42,43].

Despite reports of AEs following the booster dose, vaccination remains highly beneficial. A large study from Italy demonstrated that the bi-valent (Wild-type/Omicron BA.4–5) mRNA booster dose provided additional protection against severe COVID-19 in individuals aged 60 and older for up to six months following booster dose vaccination [44]. This protection extended even to the sub-variants BQ.1 and XBB, which were not included in the booster dose. Similarly, a study from the United Kingdom demonstrated that the bi-valent (Wild-type/Omicron BA.1) booster dose provided extra protection against hospitalization and severe disease caused by the BQ.1, CH.1.1, and XBB.1.5 sub-variants, which were also not included in the booster dose [45]. The findings from these studies suggest that the benefits of the booster vaccine outweigh the risks of forgoing it, especially for individuals at greater risk of developing severe illness.

Our study has several advantages. Specifically, our approach to collecting data regarding AEs increased the likelihood of receiving responses from individuals who would not voluntarily report AEs. It also allowed us to gather information, from individuals experiencing AEs that did not require medical care and, as a result, would not be documented in patents’ medical records.

Furthermore, while in our survey there was an almost equal distribution by sex and age group, in studies that rely on passive reporting of AEs related to the booster vaccine, the sex and age distributions varied [25,27]. Thus, our approach helps to reduce selection bias. Another advantage of our survey is the reliance on the national COVID-19 vaccination database and the national SARS-CoV-2 test results database.

This allowed us to sample the entire vaccinated adult population and validate the vaccination status and COVID-19 history of each participant. Finally, our survey collected data on AEs from the booster vaccine among people with pre-existing chronic comorbidities and compared them to previous vaccine doses.

Our survey has several limitations. First, its cross-sectional design does not allow us to determine causal relations between the booster vaccine dose and reported AEs. Second, data collected were self-reported and subjected to recall bias. Third, since this study was conducted through a telephone survey, there may be a non-response bias. Our analysis indicated that the survey respondents did not differ from non-respondents in terms of age, sex, and type of residence. However, we found that individuals with lower socioeconomic status were less likely to respond to the survey compared to those who did. Fourth, self-reported AEs and comorbidities could not be validated by reviewing respondents’ medical records, and no data were gathered regarding medication consumption. Fifth, since the purpose of our survey was to evaluate booster recipients from the general population, it did not target populations with specific underlying comorbidities, such as cancer.

## Conclusions

This survey demonstrated that most AEs following the third dose of the Pfizer BNT162b2 vaccine were transient, mild to moderate, local and general systemic reactions (i.e., fatigue, headache, fever). AEs were less prevalent among older participants aged ≥60 years and were not associated with the presence of chronic diseases, the two main target populations for COVID-19 booster vaccination. We believe the current findings may assist in lowering potential vaccine hesitancy among these populations. On the other hand, the survey showed a higher frequency of AEs among younger people and women, as well as higher frequencies of comorbidity exacerbations among individuals with underlying autoimmune or mental diseases. Therefore, counseling and monitoring for these populations are essential and recommended, as they could also help decrease potential vaccine hesitancy among them.

## Supporting information

S1 Table Outcome of telephone calls to booster vaccine dose recipients. (DOCX)

S2 Table Sociodemographic characteristics among survey participants and non-participants. (DOCX)
