# Supplementary material for: Evaluation of adverse events and comorbidity exacerbation following the COVID-19 booster dose: A national survey among randomly-selected booster recipients
Source: PLoS One. 2025 Jul 11;20(7):e0326231. doi: 10.1371/journal.pone.0326231 (PMC12250466; doi:10.1371/journal.pone.0326231)
Supplement: S1 Table — (DOCX) [file pone.0326231.s001.docx]

| **Category** | **Description** | **Sample** |
| --- | --- | --- |
| **Original sample** |  | **4,945** |
| **Not eligible** | Disconnected telephone numbers | **367** |
|  | Did not speak Hebrew | **257** |
| **Unknown eligiblity** | No connection established | 1,427 |
| **Denominator for response rate** |  | **2,894** |
| **Eligible, no interview** | Eligible respondents never available | 347 |
|  | Eligible respondents refused to participate | 469 |
| **Partial interviews** | Termination within interview/partially completed | 10 |
| **Eligible, interviewed** |  | **2,068** |
| **Interviews not used for analysis** | Inconsistent interviews | 19 |
| **Interviews completed/Final sample for analysis** |  | **2,049** |

**Table S1.** **Outcome of telephone calls to booster vaccine dose recipients**
