# Supplementary material for: Evaluation of adverse events and comorbidity exacerbation following the COVID-19 booster dose: A national survey among randomly-selected booster recipients
Source: PLoS One. 2025 Jul 11;20(7):e0326231. doi: 10.1371/journal.pone.0326231 (PMC12250466; doi:10.1371/journal.pone.0326231)
Supplement: S2 Table — (DOCX) [file pone.0326231.s002.docx]

**Table S2. Sociodemographic characteristics of the survey** **participants and non-respondents**

| **Variable** | **Non-respondents** **N=2877** **%** | **Participants**  **N=2049 %** | **P-value^1^** |
| --- | --- | --- | --- |
| **Age (Mean±SD)** | 48.3±17.0 | 47.7±16.8 | 0.21 |
| **Sex** |  |  |  |
| Male | 47.8 | 50.9 | 0.05 |
| Female | 52.2 | 49.1 |  |
| **Residence** |  |  |  |
| City | 74.0 | 75.1 | 0.08 |
| Local council | 10.5 | 13.5 |  |
| Suburban community | 15.5 | 11.4 |  |
| **Socioeconomic category status** |  |  |  |
| Low | 24.4 | 16.4 | <0.001 |
| Medium | 48.4 | 53.4 |  |
| High | 27.2 | 30.2 |  |
|  |  |  |  |

SD=Standard deviation
^1^Pearson’s Chi-square test was used for categorial variables and t-test for continuous variables
